# Molecular Epidemiology and Antimicrobial Susceptibility of Clinical *Staphylococcus aureus* from Healthcare Institutions in Ghana

**DOI:** 10.1371/journal.pone.0089716

**Published:** 2014-02-25

**Authors:** Beverly Egyir, Luca Guardabassi, Marit Sørum, Søren Saxmose Nielsen, Augusta Kolekang, Enoch Frimpong, Kennedy Kwasi Addo, Mercy Jemima Newman, Anders Rhod Larsen

**Affiliations:** 1 Department of Microbiology and Infection Control, Statens Serum Insitut, Copenhagen, Denmark; 2 Department of Veterinary Disease Biology, Faculty of Health and Medical Sciences, University of Copenhagen, Denmark; 3 Bacteriology Department, Noguchi Memorial Institute for Medical Research, Accra, Ghana; 4 Microbiology Department, University of Ghana Medical School, Accra, Ghana; 5 Department of Large Animal Sciences, Faculty of Health and Medical Sciences, University of Copenhagen, Denmark; 6 Department of Clinical Microbiology, School of Medical Sciences, Kwame Nkrumah University of Science and Technology, Kumasi, Ghana; University of Edinburgh, United Kingdom

## Abstract

The objective of this study was to determine the antimicrobial susceptibility patterns and clonal diversity of clinical *Staphylococcus aureus* isolates from Ghana. A total of 308 *S. aureus* isolates from six healthcare institutions located across Northern, Central and Southern Ghana were characterized by antibiotyping, *spa* typing and PCR detection of Panton Valentine leukocin (PVL) genes. Methicillin-resistant *S. aureus* (MRSA) were confirmed by PCR detection of *mecA* gene and further characterized by SCC*mec* and multi-locus sequence typing (MLST). The prevalence of antimicrobial resistance was below 5% for all agents tested except for penicillin (97%), tetracycline (42%) and erythromycin (6%). Ninety-one *spa* types were found, with t355 (ST152, 19%), t084 (ST15, 12%) and t314 (ST121, 6%) being the most frequent types. Based on established associations between *spa* and MLST types, isolates were assigned to 16 clonal complexes (CCs): CC152 (n = 78), CC15 (n = 57), CC121 (n = 39), CC8 (n = 36), CC5 (n = 33), CC1 (n = 29), CC45 (n = 9), CC88 (n = 8), CC30 (n = 4), CC9 (n = 3), CC25 (n = 2), CC97 (n = 2) CC20 (n = 2), CC707 (n = 2), CC7 (n = 3) and CC522 (n = 1). Most isolates (60%) were PVL-positive, especially those belonging to ST152, ST121, ST5, ST15, ST1, ST8, and ST88. Nine (3%) isolates were MRSA belonging to seven distinct clones: ST88-IV (n = 2), ST250-I (n = 2), ST8-IV (n = 1), ST72-V (n = 1), ST789-IV (n = 1), ST2021-V (n = 1), and ST239-III (n = 1). The study confirmed a high frequency of PVL-positive *S. aureus* in Africa, low prevalence of antimicrobial resistance and high diversity of MRSA lineages in Ghana compared to developed countries and other African countries. The detection of known pandemic MRSA clones in the absence of routine MRSA identification in most Ghanaian clinical microbiology laboratories calls for capacity building to strengthen surveillance and prevent spread of these clones.

## Introduction

Methicillin-resistant *Staphylococcus aureus* (MRSA) is a major concern in clinical medicine due to the importance of β-lactams in the therapy of staphylococcal infections and the additional morbidity and mortality for MRSA patients compared to patients infected with methicillin-susceptible *S. aureus* (MSSA) [1]. Despite the importance of MRSA, MSSA are among the most common causative agents of bacteraemia and skin and soft tissue infections (SSTI) [2]. Epidemiological surveillance of MRSA and MSSA is of importance for the development and implementation of infection control programmes. Data on *S. aureus* epidemiology in African countries are limited and a common trait for MSSA strains from various African countries seems to be the carriage of the PVL genes: *lukS/F-pv* at much higher frequencies (>55%) than in the rest of the world (<10%) [2]–[6]. The high frequency of PVL among human MSSA strains is of special interest since the most successful community associated (CA) MRSA clones share this genetic marker, and could have MSSA ancestors associated with Africa as recently suggested [5], [6]. PVL is associated with SSTI and severe necrotising pneumonia and has been shown to be a characteristic feature of community acquired (CA) -MRSA clones disseminated in Europe and Middle East (ST-80), Australia and South America (ST30-IV), and United States (ST8-IV, also known as USA300) [2], [7], [8].

The objective of this study was to investigate the antimicrobial susceptibility and clonal diversity of clinical *S. aureus* isolates from Ghana. Antimicrobial resistance in *S. aureus* has previously been reported from Ghana with findings of a low MRSA prevalence in nasal swabs from patients and health care workers at the Korle-bu Hospital, Accra [9] however, treatment in Ghana is mainly empirical due to a relative lack of appropriate laboratory facilities [10] and therefore only few susceptibility data exists and so far no study has investigated the clonal structure of *S. aureus* in clinical samples. The study was part of a cooperation program on Antibiotic Drug use, Monitoring and Evaluation of Resistance (ADMER) in Ghana under the Danish Ministry of Foreign Affairs. This program was conceived to strengthen clinical microbiology and surveillance of antibiotic resistance, and ultimately to improve awareness of antimicrobial use in Ghana.

## Materials and Methods

### Ethics Statement

Ethical clearance was obtained from the University of Ghana Medical School Ethical and Protocol Review Board (reference no. MS-EI/M.9 - P.3.212010-11).

### Bacterial Isolates

**Figure 1:**
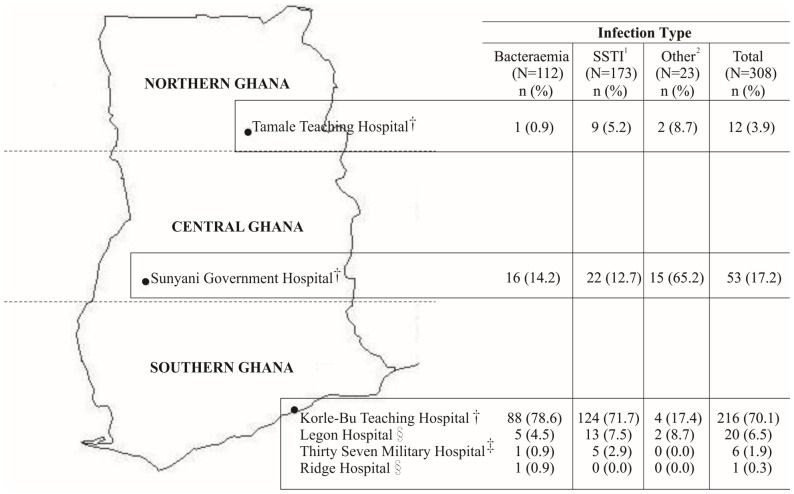
Origins of the 308 clinical *Staphylococcus aureus* collected from six hospitals in Ghana, 2010–2012. †Referral Hospitals. ‡Secondary Hospitals. §Primary Hospitals. ^1^SSTI: Skin and Soft Tissue Infections. ^2^Other: (Urinary Tract Infection (n = 9); Unknown Infections (n = 14)).

Staphylococcal isolates from clinical specimens were obtained in a prospective cross-sectional-like study between October 2010–June 2012 from six healthcare institution situated at Northern (Tamale Teaching Hospital), Central (Sunyani Government Hospital) and Southern Ghana (Korle bu Teaching Hospital, Thirty-seven Military Hospital, Ridge Hospital and Legon Hospital) (Figure 1). The majority of the isolates (70%) were obtained from Korle bu Teaching Hospital, which serves a population of over 3 million and acts as a major referral health facility for an estimated population of 24 million people across Ghana. Presumptive staphylococci identified by colony morphology at the hospital clinical microbiology laboratories were collected and sent to Noguchi Memorial Institute for Medical Research, where they were identified as *S. aureus* by Gram staining, catalase, tube coagulase and slidex staphplus test (bioMérieux, Marcy l’Etoile, France). Available patient demographic characteristics such as age and sex were retrieved from laboratory records.

### Antimicrobial Susceptibility Testing

Susceptibility testing was carried out by disc diffusion technique following the European Committee on Antimicrobial Susceptibility Testing (EUCAST) guidelines (www.eucast.org) using 1U penicillin, 30 µg tetracycline, 30 µg cefoxitin, 2 µg clindamycin, 15 µg erythromycin, 10 µg norfloxacin, 10 µg gentamicin, 10 µg linezolid, 5 µg rifampicin, 1. 25 µg +23.75 µg trimethoprim-sulfamethoxazole, and 10 µg fusidic acid (Rosco NeoSenstabs, Taastrup, Denmark). Inducible clindamycin resistance was detected by placing clindamycin and erythromycin 12–20 mm apart (D-test). Brain Heart Infusion agar supplemented with teicoplanin (5 mg/L) (Becton Dickinson, Denmark) was used to screen MRSA isolates for glycopeptides resistance by a spot test; if 10 or more colonies were detected on these plates, E-tests (bioMérieux, Marcy I’Etoile, France) were used to determine the minimum inhibitory concentration of vancomycin and teicoplanin [11]. Multidrug resistance (MDR) was defined as resistance to at least three distinct antimicrobial classes or being MRSA [12].

### Molecular Typing

Molecular characterization of the isolates was done at Statens Serum Institut (SSI), Denmark. A multiplex PCR was used for detection of *spa*, *lukS/F-pv* and *mecA* [13]. *spa* typing was performed as described by Harmsen et al. [14]. Using BioNumerics v.6.5 (Applied Maths, Sint-Martens-Latem, Belgium) with the Ridom *spa* server (http://spa.server.ridom.de) plug-in, *spa* sequences were automatically assigned to *spa* types and clonal complexes (CCs) based on *spa* repeats. Multi-Locus Sequence Typing (MLST) [15] was done on all MRSA and MSSA isolates whose CC could not be assigned by the Ridom *spa* server. Minimum Spanning Tree (MST) based on *spa*- types was made using BioNumerics V6.5 (Applied Maths, Sint-Martens-Latem Belgium). Staphylococcal cassette chromosome *mec* (SCC*mec*) typing was performed by multiplex PCR as described previously [16].

### Statistical Analysis

Distributions of the various genotypes determined in the study (PVL-positivity, *spa* type, ST and CC) were associated to region, hospital, sex and infection type to determine if specific patterns existed. Only genotypes with more than 10 observations were included in statistical analysis. MRSA isolates were not evaluated due to the low prevalence of their genotypes. Associations were determined using the χ^2^ test, except for PVL-positivity, which was analysed by logistic regression. A significant association was deemed at p-values <0.05.

## Results

Of the 903 presumed staphylococci collected from the six hospitals, 308 (34%) were identified as *S. aureus* and 595 (66%) as coagulase negative staphylococci. *S. aureus* isolates originated from SSTI (n = 173), bacteraemia (n = 112), and other (urinary tract infection, n = 9; unknown infections, n = 14) infections (n = 23). *S. aureus* was isolated from 143 females and 109 males. Sex origin of 56 isolates could not be traced from laboratory records. With regard to hospital origin, 12 isolates were from Tamale Teaching Hospital (TTH, Northern Ghana), 53 from Sunyani Government Hospital (SGH, Central Ghana) and 243 from the four hospitals in Southern Ghana. Details of hospital location (stratified into regions of study) and proportions of isolates from clinical infections are shown in Figure 1. None of the clinical laboratories used methods for MRSA detection and typing, and several pitfalls were recognized in routine microbiological procedures (e.g. poor identification to species/genus level, and low compliance with international standards for susceptibility testing).

The highest prevalence of resistance was for penicillin (97%), followed by tetracycline (42%) and erythromycin (6%). Lower percentages of resistance were observed for clindamycin (5%), norfloxacin (4%), trimethoprim-sulphamethoxazole (4%), gentamicin (3%), cefoxitin (3%) and fusidic acid (2%). Inducible clindamycin resistance was detected among seven (2%) isolates. Twenty-nine isolates (9%) were MDR, of which 9 (3%) were confirmed *mecA* positive MRSA. Details of MDR isolates have been shown in Table 1. MRSA isolates were susceptible to vancomycin and teicoplanin. Most of the MSSA isolates (88%, 264/299) were resistant to penicillin (n = 154), penicillin and tetracycline (n = 99) and penicillin and trimethoprim-sulphamethoxazole (n = 11). All isolates were susceptible to linezolid and rifampicin while three isolates (1%) were susceptible to all antimicrobial drugs tested.

**Table 1:** Origins and characteristics of 29 multi-drug resistant (MDR) *Staphylococcus aureus* isolated from healthcare institutions in Ghana, 2010–2012.

	ID	Hospital^a^	Infection^b^	CC	ST	*spa* type	SCC*mec*	PVL	Antibiotype^c^
MRSA	5016	KB	SSTI	CC1	ST72	t537	V	−	Fox, Pen, Tet
	744	KB	Blood	CC8	ST2021	t024	V	−	Fox, Pen, Tet
	2244	KB	Blood	CC8	ST239	t037	III	−	Fox, Pen,Tet, Fuc, Gen, Cli, Ery,
	3464	KB	Blood	CC8	ST8	t121	IV	+	Fox, Pen, Nor, Cli, Ery
	2207	KB	SSTI	CC8	ST250	t928	I	−	Fox, Pen, Tet, Gen, Nor, Cli, Ery
	2224	KB	SSTI	CC8	ST250	t928	I	−	Fox, Pen, Tet, Gen, Nor, Cli, Ery
	44	SGH	Unknown	CC88	ST88	t186	IV	−	Fox, Pen, Tet
	AU81	SGH	SSTI	CC88	ST88	t186	IV	−	Fox, Pen, Tet
	11087	KB	UTI	CC152	ST789	t547	IV	+	Fox, Pen, Tet, Nor
MSSA	2639	KB	Blood	CC1	ST1	t7835	NA	+	Pen, Tet, Cli, Ery
	AU93	SGH	SSTI	CC1	ST1	t559	NA	+	Pen, Tet, Fuc
	A6	KB	Unknown	CC5	ST5	t311	NA	+	Pen, Tet, Fuc
	5095	KB	SSTI	CC5	ST5	t071	NA	+	Pen, Tet, Gen, TMS, Fuc, Nor, Cli, Ery
	T2845	TTH	SSTI	CC8	ST8	t451	NA	+	Pen, Tet, TMS
	1455	KB	SSTI	CC9	ST9	t2700	NA	−	Pen, Gen, Cli, Ery
	1050	KB	Blood	CC15	ST15	t084	NA	+	Pen, Tet, Gen
	2320	KB	Blood	CC45	ST508	t635	NA	−	Pen, Cli, Ery
	1548	KB	SSTI	CC88	ST88	t10809	NA	+	Pen, Tet, Nor, Cli, Ery
	5270	KB	SSTI	CC88	ST88	t10810	NA	+	Pen, Tet, Nor, Cli, Ery
	NAB	KB	SSTI	CC121	ST121	t213	NA	_	Pen, Tet, Fuc
	3209	KB	Blood	CC121	ST121	t091	NA	_	Pen, Tet, Nor, Gen, Cli, Ery
	2437	KB	Blood	CC121	ST121	t091	NA	_	Pen, Tet, Nor
	5293	KB	Blood	CC121	ST121	t314	NA	+	Pen, Tet, Nor
	5775	KB	Blood	CC121	ST121	t314	NA	+	Pen, Tet, Nor
	3984	KB	SSTI	CC152	ST152	t1299	NA	+	Pen, Tet, Cli, Ery
	1544	KB	SSTI	CC152	ST152	t355	NA	+	Pen, Tet, Cli, Ery
	4836	KB	SSTI	CC152	ST152	t355	NA	+	Pen, Tet, Cli, Ery
	112242	MH	SSTI	CC152	ST152	t355	NA	+	Pen, Tet, Ery
	A71	SGH	Blood	CC152	ST152	t355	NA	+	Pen, Tet, Fuc

ST: Sequence Type; CC: Clonal Complex; SCC: Staphylococcal Cassette Chromosome; PVL: Panton- Valentine leukocidin.

a KB: Korle bu Teaching Hospital; SGH: Sunyani Government Hospital; TTH: Tamale Teaching Hospital; MH: Military Hospital.

b SSTI: Skin and Soft Tissue Infection; UTI: Urinary Tract Infection; ^c^ TMS: trimethoprim-sulphamethoxazole.

c Pen: penicillin; Fox: cefoxitin; Tet: tetracycline; Nor: norfloxacin; Gen: gentamicin; Fuc: Fucidic acid; Cli: clindamycin; Ery: erythromycin.

**Figure 2:**
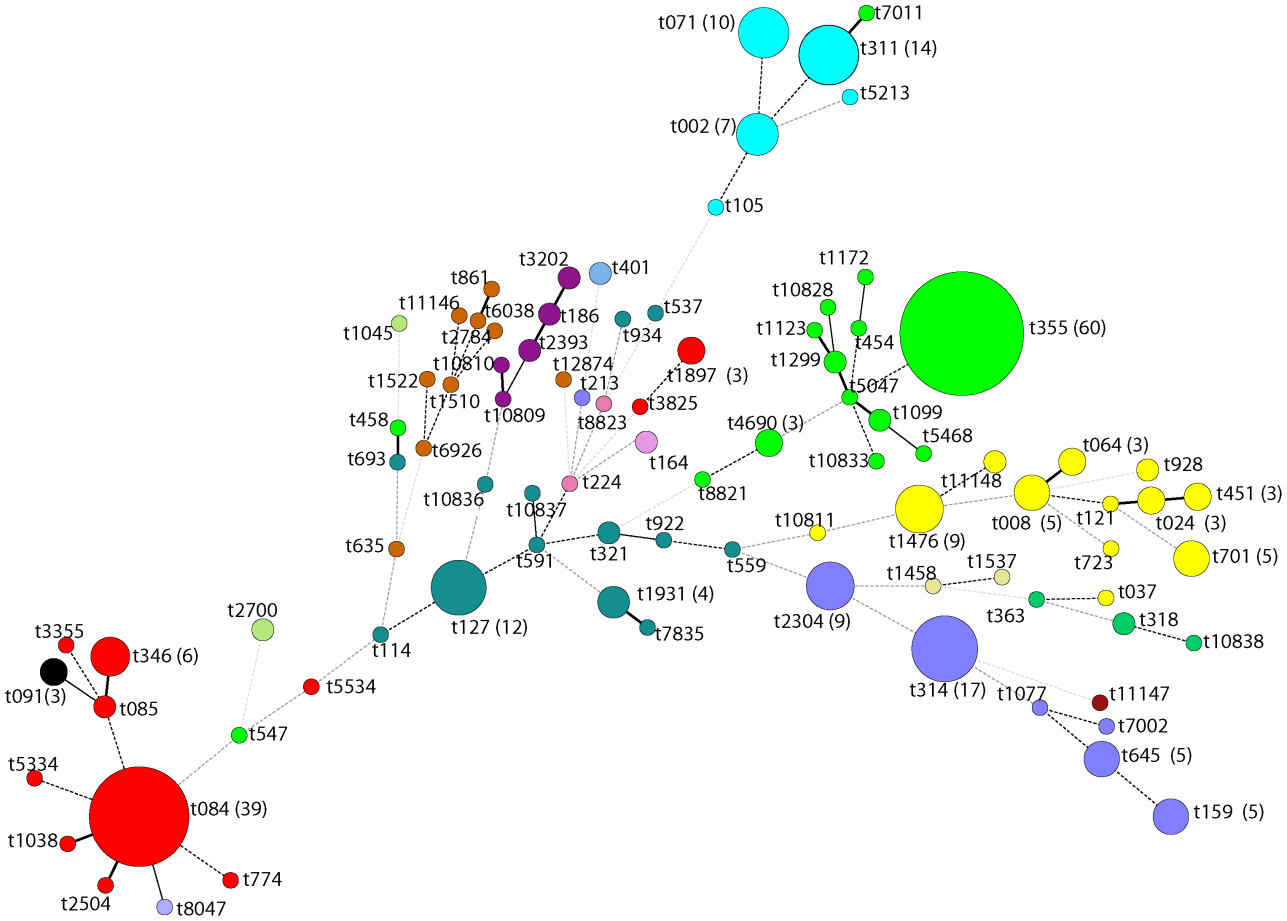
Minimum spanning tree of 308 clinical *Staphylococcus aureus* isolates from healthcare institutions in Ghana. Nodes indicate *spa* types and their size shows the relative number of isolates for each *spa* type. Numbers of frequent (three or more) *spa* types have been shown. Every colour represents a distinct clonal complex.

High genetic diversity was observed by *spa* typing, as indicated by the recovery of 91 *spa* types among all isolates tested. The most common *spa* types were t355 (19%), t084 (12%), t314 (6%) and t311 (5%). Fifty-six *spa* types were singletons and eight new *spa* types were detected: t10809 (ST88), t10810 (ST88), t10811 (ST8), t10828 (ST152), t10833 (ST152), t10836 (ST1), t10837 (ST1) and t10838 (ST30). A minimum spanning tree, including *spa* types and clonal complexes of the 308 isolates is shown in Figure 2.

Based on *spa* typing, isolates (n = 308) were assigned to 16 MLST clonal complexes: CC152 (n = 78), CC15 (n = 57), CC121 (n = 39), CC8 (n = 36), CC5 (n = 33), CC1 (n = 29), CC45 (n = 9), CC88 (n = 8), CC30 (n = 4), CC9 (n = 3), CC7 (n = 3), CC25 (n = 2), CC97 (n = 2) CC20 (n = 2), CC707 (n = 2), and CC522 (n = 1). MRSA isolates belonged to ST88-IV (n = 2), ST8-IV (n = 1), ST789-IV (n = 1), ST72-V (n = 1) ST2021-V (n = 1), ST250-I (n = 2), and ST239-III (n = 1). Two of them were PVL-positive and belonged to t121 (ST8) and t547 (ST789) (Table 1). The most common MSSA lineages were ST152 (27%) and ST15 (18%).

Five *spa* types (t084, t127, t311, t314 and t355), six STs (ST1, ST5, ST8, ST15, ST121 and ST152) and six CCs (CC1, CC5, CC8, CC15, CC121 and CC152) were included in the statistical analysis. Surprisingly, s*pa* type t311 occurred less frequently among females (0.3%) than among males (2%) and those of unknown sex (2.3%) (p = 0.0016). No other clinical or spatial associations were observed in the distribution of *spa-*types, STs and CCs with regard to infection type, sex, region and hospital of origin, Some spatial variations were observed in the distribution of *spa* types (e.g. t355 occurred in 34% and 13% isolates from Sunyani Government Hospital and Korle bu Teaching Hospital respectively) but such differences could not be proven to be significant. Isolates from Ridge Hospital (n = 1), Thirty-seven Military Hospital (n = 6) and Tamale Teaching Hospital (n = 12) were excluded from the statistical analysis due to low numbers.

PVL was detected in 60% (n = 184) of the isolates, mainly isolates from SSTIs (57%) and belonging to ST152 (38.5%), ST121 (21%), ST5 (13.5%), ST15 (11%), ST1 (7%), ST8 (3.8%) and ST88 (2.7%). Genotypes and clinical origins of PVL positive *S. aureus* are shown in Table 2. The patterns of PVL varied by region, with Central regions having a higher chance 2.2 (95% CI: 1.1–4.2) of seeing PVL-positive *S. aureus* (p = 0.02) than the Southern region.

**Table 2:** Clonal complex (CC), multi-locus sequence type (ST), *spa* type and clinical origin of 184 *Staphylooccus aureus* harbouring Panton-Valentine leukocidin (PVL) genes isolated in Ghana, 2010–2012. STs and CCs were inferred from *spa* types.

CC	ST	*spa* type (N)	Clinical origin, N (%)				
			Bacteraemia N = 65	SSTI N = 104		Other* N = 15	TotalN = 184
CC1	ST1	t127 (3), t1931 (3), t693 (1), t559 (1), t10836 (1),t114 (1), t922 (1), t934 (1), t7835 (1)	6 (9.2)	7 (6.7)		0 (0.0)	13 (7.0)
CC5	ST5	t071 (9) t311 (9), t002 (5), t105 (1)	12 (18.5)	11 (10.6)		1 (6.7)	24 (13.0)
CC8	ST8	t1476 (3), t024 (1), t451 (1), t064 (1), °t121(1)	2 (3.0)	5 (4.8)		0 (0.0)	7 (3.8)
CC15	ST15	t084 (19), t5534 (1), t774 (1)	7 (10.8)	13 (12.5)		1 (6.7)	21 (11.4)
CC25	ST25	t401 (1)	1 (1.5)	0 (0.0)		0 (0.0)	1 (0.5)
CC30	ST30	t10838 (1),t363 (1)	0 (0.0)	2 (1.9)		0 (0.0)	2 (1.1)
CC88	ST88	t2393 (2) t3202 (1), t10809 (1), t10810 (1)	1 (1.5)	4 (3.8)		0 (0.0)	5 (2.7)
CC121	ST121	t314 (15), t2304 (9), t159 (5), t645 (5), t1077(1), t7002(1)	14 (21.5)	21 (20.2)		1 (6.7)	36 (19.6)
CC152	ST152	t355 (57), t4690 (3), t1096 (2), t1299 (2), Singletons (11)^a^	22 (34.0)	41 (39.4)		12 (80.0)	75 (40.8)

SSTI: Skin and Soft Tissue Infection;*Other: UTI: Urinary Tract Infection (n = 5: *spa* types t355 (3), t547(1) and t5534 (1); Unknown (n = 10; *spa* types: t311 (1), t645 (1), t4690 (1), t355 (7)^ a^Other *spa* types associated with CC152: t454, t458, t5268, °t547, t1123, t1172, t5047, t7011, t8821, t10828, and t10833°MRSA.

## Discussion

This study fills an important gap in the knowledge of the epidemiology of *S. aureus* in Ghana. As such, the study contributes to the current knowledge of the diversity and population structure of this important bacterial pathogen at the global level. Ghana and several other African countries have so far been black spots on the map due to lack of established national surveillance programmes and adequate clinical microbiology infrastructure [10], [17]. Our results show that the most common *spa t*ypes among MSSA isolates are t355 (ST152) and t084 (ST15). The *spa* types were previously found to be predominant among *S. aureus* isolates from asymptomatic nasal carriers at Korle bu, the largest Teaching Hospital in Ghana [9], suggesting that they are well established in the human population of this country. In another African study, t084 (ST15) was also reported as one of the most frequent *spa* types among *S. aureus* isolated from seven tertiary hospitals located in five major African towns [3]. PVL-positive ST152 (t355) is also widely distributed in African countries [4], [5] and its frequent recovery from SSTI is consistent with studies in other countries [4], [18]. Other PVL-positive MSSA lineages found in this study such as ST121, ST30, ST15 and ST5 have also been reported elsewhere in Africa [19]. The observed high prevalence (60%) of PVL appears to be a distinguishing genetic trait of African MSSA [3], [4], [6] compared to USA, Asia and Europe, where this virulence factor is uncommon in MSSA [2], [19], [20]. This finding was correlated to the high frequency of PVL-positive ST152, which is a likely ancestor of the CA-MRSA ST152-V clone circulating in certain European regions, especially the Balkan area [21], [22].

The nine MRSA isolates belonged to seven unrelated *spa* types and STs harbouring four different SCC*mec* types (Table 1), indicating high clonal diversity. Some of the MRSA lineages identified in this study are widely distributed worldwide: ST239-III is a pandemic clone prevalent in Europe, Asia and South Africa [23]–[25] and ST789-IV is a single locus variant of the ST7 clone frequently reported in Asia [25]. ST88-IV, ST8-IV and ST72-V have been previously reported among inpatients and staff at Korle-bu Hospital in Ghana [9] and in communities and hospitals in other African countries [26], [27]. MRSA ST88 has been reported sporadically in some European countries like Portugal [28] and Sweden [29]. ST8-IV MRSA (*spa* type t121, PVL+) found in this study is related to the epidemic MRSA ST8-IV (USA300) clone in the USA [2]. Other African studies have reported this ST8-IV MRSA (*spa* type t121, PVL+) strain in communities and hospitals [26], [30]. ST250-I, also referred to as the “Archaic clone”, differs from ST8 by a point mutation in the *yqiL* gene and is related to ST247-I (Iberian clone), a major clone isolated in European hospitals [7], [31]. ST72 has been reported as a major MRSA clone from communities in Australia [32] and as MSSA in Nigeria and Gabon [6], [27]. The least known MRSA lineage found in this study was ST2021-V, which to the best of our knowledge has previously been reported in a single isolate from Nigeria (www.mlst.net; accessed on: 4^th^ April 2013). Although ST5, ST30 and ST80 MRSA have been described in several African and other countries around the world [26], [27], none of these clones were detected among clinical MRSA isolates in Ghana. PVL-positive ST5 and ST30 were however detected among MSSA isolates (Table 2), indicating that these two *S. aureus* lineages are widespread in African countries, even though acquisition of methicillin resistance seems to be confined to some countries.

The prevalence of antimicrobial resistance in clinical *S. aureus* isolates from Ghana was generally low. Other African studies have reported similar levels of resistance to penicillin (86%–93%) and tetracycline (28%–48%) but higher levels of resistance to sulphonamides (22%–68%) compared to this study [3], [4], [9]. Comparatively, the prevalence of MRSA (3%) was lower than those reported in other African countries such as Nigeria (20%) [27], Algeria (45%) [33] and in a multicenter study (15%) involving five major African towns [26]. The low MRSA frequency reported in this study could be attributed to the low consumption of antimicrobial agents such as fluoroquinolones and third generation cephalosporins in Ghana, because they are expensive and are usually prescribed for acute infections [10]. Usage of the afore-mentioned antimicrobial agents has been shown to correlate with an increase in MRSA prevalence [34]–[36]. The observed MRSA prevalence among clinical isolates in Ghana is similar to those reported in European countries with low MRSA prevalence, such as the Scandinavian countries and The Netherlands [37].

Some apparent geographical variations in clonal distribution were observed, but the low number of isolates obtained from the Northern region made comparisons between hospitals or regions meaningless. The clinical information on the 308 *S. aureus* included in the study varied in quality due to incompleteness of the patient records collected from the various hospital clinical laboratories involved in the study. Thus, it was not possible to determine possible associations between antimicrobial therapy and resistance patterns.

We conclude that MRSA occurs at low prevalence among *S. aureus* investigated in this study. MRSA clones circulating in the country are genetically diverse and a number of them belong to known pandemic clones. The overall levels of antimicrobial resistance are generally low compared to other African countries and to most developed countries, most likely because of the low usage of antimicrobial agents in the country. On the other hand, the study also denotes absence of routine MRSA testing and poor performance standards in most clinical microbiology laboratories in Ghana, highlighting the need for infrastructures to support national antimicrobial policies and surveillance capacity.
